# Anemia Burden among Hospital Attendees in Makkah, Saudi Arabia

**DOI:** 10.1155/2022/4709119

**Published:** 2022-04-22

**Authors:** Ahmad Fawzi Arbaeen, Mohammad Shahid Iqbal

**Affiliations:** Faculty of Applied Medical Sciences, Department of Laboratory Medicine, Umm Al-Qura University, Makkah Al Mukarramah, Saudi Arabia

## Abstract

**Background:**

Anemia is a major health problem in Saudi Arabia and has multiple etiologies. Many studies have been conducted in Saudi Arabia in specific population groups like school children, adolescents, university students, and females in the reproductive age group, and most have reported high prevalence of anemia. This study was conducted in a specialist hospital in Makkah city and includes all outpatients aged 15 years and above.

**Objective:**

To study the burden of anemia among hospital attendees, its stratification based on gender and age, and its severity along with the morphological types of anemia.

**Methods:**

This is a study conducted at a specialist hospital in Makkah city and one-month data were collected retrospectively from the laboratory database and include demographic and routine hematological results of complete blood count (CBC).

**Results:**

A total of 21,524 patients were included, out of which 9444 (43.9%) were males and 12020 (56.1%) were females. The overall prevalence of anemia was 38.7% (8339). Prevalence was very high in females, accounting for 68.2% (5689), whereas it was 31.8% (2650) in males. There were 39.6% (3301), 43.9% (3657), and 16.6% (1381) cases of mild, moderate, and severe anemia, respectively. In females, anemia was more prevalent in the age group of 15 to 49, which is considered as the reproductive age group. Microcytic anemia was the most prevalent type observed in this age group, accounting for 40.7% of all anemia cases. Normocytic anemia was more prevalent in the males, accounting for 52%.

**Conclusion:**

Our study showed high prevalence of anemia among the patients attending outpatient departments in a specialist hospital. Females have high prevalence of anemia when compared to male population. Microcytic anemia was the most common anemia type among females and was seen in the 15–49 age group. There is an increase in prevalence of anemia with age for males, whereas, in females, increased prevalence is observed in the reproductive age groups and the anemia prevalence maintained a steady decrease towards the 5th to the 9th decades. Normocytic anemia was more prevalent in the 5th to the 9th decades, indicating that there are more etiologies other than iron deficiency in the causation of anemia. Macrocytic anemia was the least reported anemia type. Anemia of mild and moderate severity was predominant in both genders, although severe anemia showed higher prevalence in females as compared to males.

**Conclusion:**

Our study showed high prevalence of anemia among the patients attending outpatient departments in a specialist hospital. Females have high prevalence of anemia when compared to male population. Microcytic anemia was the most common anemia type among females and was seen in the 15–49 age group. There is an increase in prevalence of anemia with age for males, whereas, in females, increased prevalence is observed in the reproductive age groups and the anemia prevalence maintained a steady decrease towards the 5th to the 9th decades. Normocytic anemia was more prevalent in the 5th to the 9th decades, indicating that there are more etiologies other than iron deficiency in the causation of anemia. Macrocytic anemia was the least reported anemia type. Anemia of mild and moderate severity was predominant in both genders, although severe anemia showed higher prevalence in females as compared to males. *Conclusion*. Anemia is highly prevalent in adolescents, adults, and the elderly in Makkah region. The most common cause is thought to be iron deficiency, although other causes are not uncommon. The authorities need to address the problem of prevention and reduction in anemia prevalence by taking effective measures and interventions.

## 1. Introduction

Anemia is a global health issue affecting a quarter of the global population [1]. It affects almost every country and occurs in all age groups but is more prevalent during pregnancy and childhood [2]. In many developing countries, anemia has reached the level of an epidemic [3]. It is reported that half of the cases of anemia are due to iron deficiency, which is the most common micronutrient deficiency [4, 5]. Along with nutritional deficiencies, malaria, parasitic infections, blood loss, hemoglobinopathies, and bone marrow suppression or replacement are common etiologies [6]. Anemia is defined by WHO as a condition in which hemoglobin concentration is below 120 g/L in nonpregnant females and below 130 g/L in males [2]. Iron deficiency causes chronic fatigue and tiredness and can affect cognitive functions, as well as motor and mental development along with visual and auditory functions of people [7]. Anemia is found in varying degrees across the world, depending on the age group and geographic location. Anemia affects one out of every four people, with pregnant women and children under the age of five being the most vulnerable. With two-thirds of preschool-aged children and half of all women impacted, the WHO areas of Africa and Southeast Asia are the most at risk [2]. In terms of numbers, the majority of the burden is concentrated in South-East Asia, where roughly 40% of anemic preschool-age children and nonpregnant women, as well as approximately 30% of pregnant women, reside [8].

WHO has reported global prevalence of 30.2% among nonpregnant women (15–49.99 years) and about 33% in Asia and 44.4% in Africa. Among men (aged 15–59.99 years) and the elderly (≥60 years), the global anemia prevalence is 12.7% and 23.9%, respectively [2].

Anemia is a common problem in low- and middle-income countries, particularly among adolescent females, women of reproductive age, pregnant women, and children. Anemia is expected to be reduced in women of reproductive age (15–49 years) by 50 percent by 2025, according to the second of the world's six global nutrition objectives. Given the fact that anemia affects half a billion women of reproductive age around the world, eliminating anemia is essential for the health as well as their economic production. According to the Global Health Observatory, anemia prevalence among women of reproductive age ranged from 9.1 percent in Australia to 69.6 percent in Yemen in 2016 [8].

According to reports, anemia is a significant health burden in the Gulf countries, with a high frequency of anemia in females between the ages of 17 and 24 years, as well as males, being reported [4]. Preschool children, pregnant women, and nonpregnant women all have high prevalence of anemia in Saudi Arabia, which, according to the World Health Organization's report on the worldwide prevalence of anemia, is considered a moderate health problem in the country, with prevalence ranging between 20.0 and 39.9 percent [2].

According to the findings of a study conducted on Saudi women aged 15–49 years, anemia was found in 40% of the participants [9]. Another investigation by Al Quaiz found a significant frequency of anemia among females from Riyadh, with an estimated 37 percent of females suffering from the condition [7]. Many studies have been undertaken in various population groups such as school children, teenagers, university students, and females in the reproductive age group in Saudi Arabia, and the findings have revealed a high incidence of anemia in these categories in the country [5, 10–13]. In this study, we aimed to determine the prevalence of anemia, its stratification based on gender, its severity, and the morphological type of anemia in all of the study patients.

## 2. Materials and Methods

This study was done using the patient's data collected retrospectively for one month from a specialist hospital in Makkah city. The data were collected from the laboratory database and include demographic and routine hematological results of complete blood count (CBC) performed on a fully automated hematology analyzer. Hematological data from all patients aged 15 years and above and attending the routine outpatient departments over a period of one month from January 1, 2019, to January 31, 2019, were included for analysis.

The hemoglobin cut-off (g/L) for the diagnosis of anemia and its categorization based on severity into mild, moderate, and severe anemia was done as per WHO recommendation as shown in Table 1 [14].

Statistical Analysis was done using IBM SPSS Statistic (Statistical Package for the Social Sciences, version 20, Armonk, New York, USA). *P* value <0.05 was considered statistically significant. Continuous variables were expressed as mean ± standard deviation and categorical data were presented as median and interquartile range (IQR). Odds ratios (ORs) and 95% confidence intervals were obtained by logistic regression to determine the impact of age on anemia.

## 3. Results

**Figure 1:**
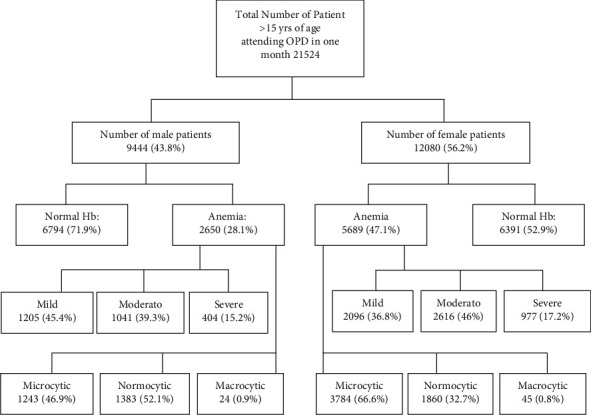
Flow chart showing the patient selection and stratification.

**Figure 2:**
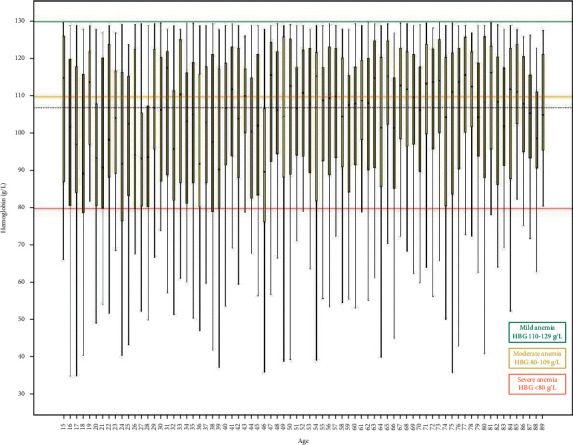
Anemia groups stratified based on hemoglobin concentration for male gender and age.

**Figure 3:**
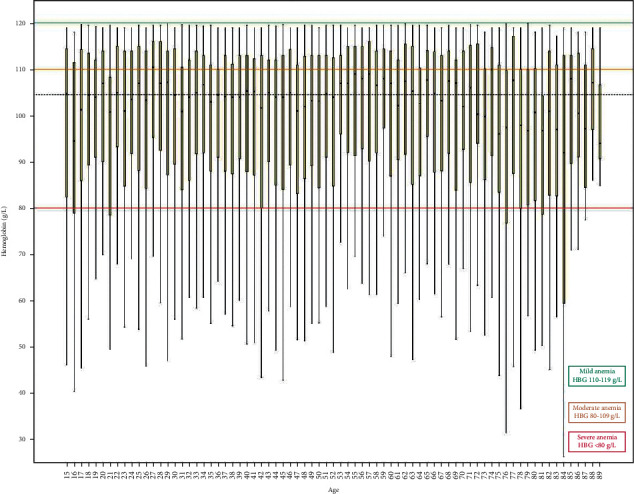
Anemia groups stratified based on hemoglobin concentration for female gender and age.

The specialist hospital lab receives between 400 and 500 blood samples for routine hematological tests every day from patients attending the outpatient clinics. A total of 21,524 patients were included, out of which 9444 (43.87%) were males and 12020 (56.2%) were females (Figure 1). The mean age for male patients was 48.83 ± 18.7, with a range of 15 to 89 years, and for females it was 46.88 ± 17.8, with a range of 15 to 88 years. We included 21,524 hemoglobin estimations and none of the patients had repeated hemoglobin value. The overall prevalence of anemia was 38.7% (8339). Among all the anemia cases, prevalence was very high in females, accounting for 68.2% (5689), whereas it was 31.8% (2650) in males.  Table 2 shows the hematological variables for male and female patients. Overall, there were 39.6% (3301), 43.9% (3657), and 16.6% (1381) cases of mild, moderate, and severe anemia, respectively. The mean hemoglobin concentration was 125.29 ± 25.7. The mean hemoglobin in males was 100.12 ± 19.2 g/l and in females it was 98.8 ± 18.2 g/l. Tables 3 and 4 along with Figures 2 and 3 present the age-wise distribution of hemoglobin and stratification into mild, moderate, and severe anemia based on WHO recommendation for males and females, respectively [14].

**Figure 4:**
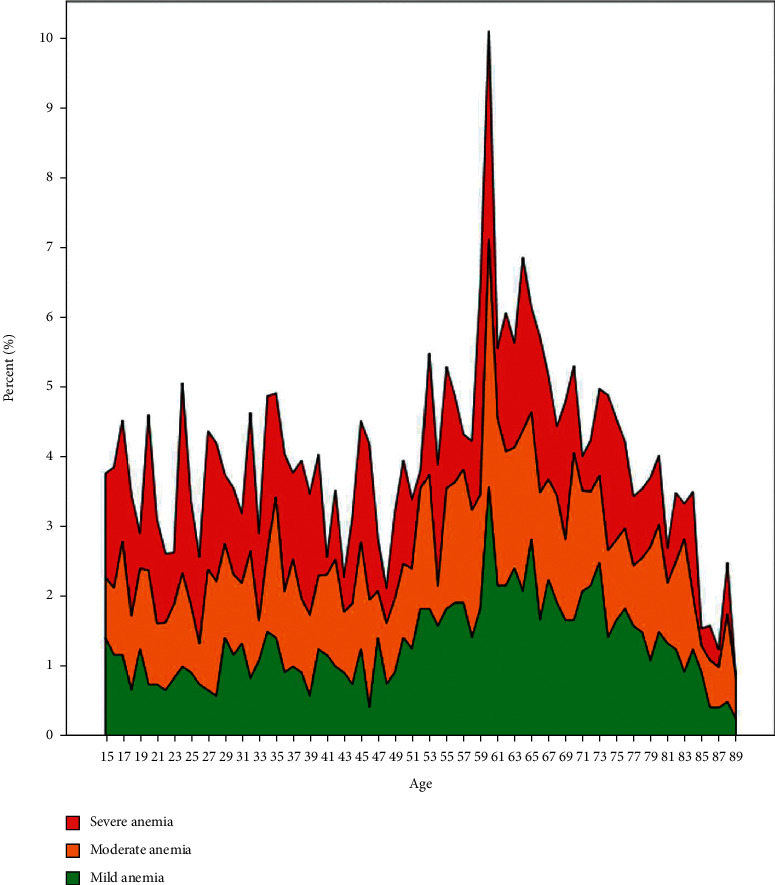
Age-wise prevalence of mild, moderate, and severe anemia in males.

**Figure 5:**
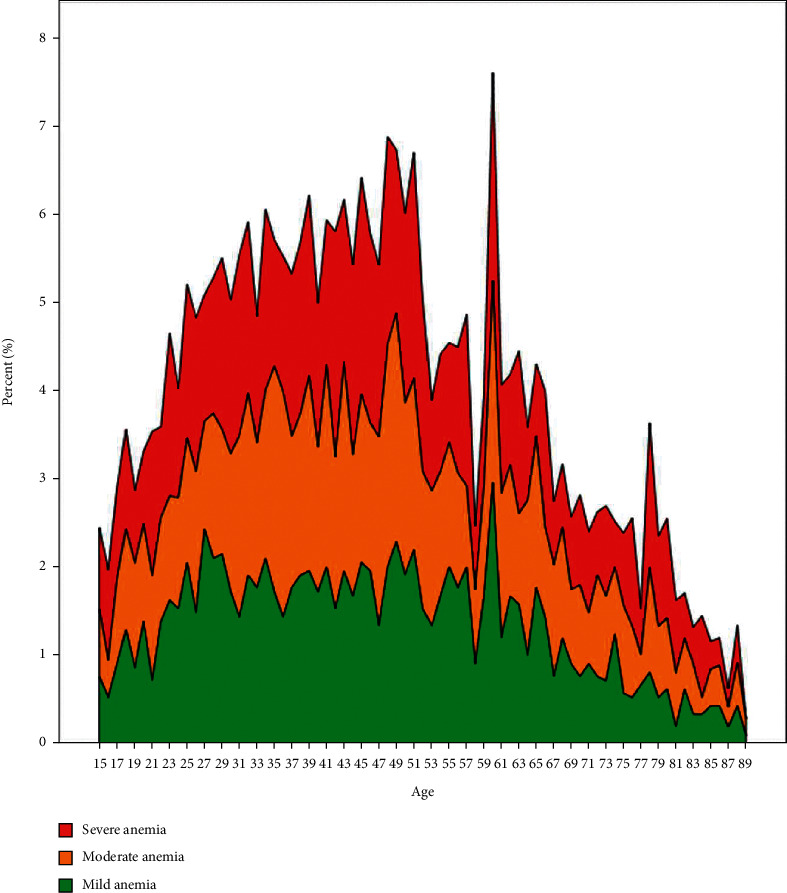
Age-wise prevalence of mild, moderate, and severe anemia in females.

All the anemia cases were categorized into microcytic, normocytic, and macrocytic anemia based on the reference cut-off for mean corpuscular volume (MCV). Microcytic anemia was the most prevalent type among females, whereas normocytic anemia was more prevalent in the males, accounting for 52% of the anemia cases in males (Tables 5 and 6; Figures 4 and 5). The prevalence of macrocytic anemia was very low in both genders. Among the male patients, high prevalence of mild and moderate anemia is observed in all age groups, although the 6th, 7th, and 8th decades showed increased prevalence, whereas severe anemia was distributed uniformly over all the age groups. In females, anemia was more prevalent in the age group of 15 to 49 which is considered as the reproductive age group. Microcytic anemia was the most prevalent type observed in this age group, accounting for 40.7% of all anemia cases (Table 6). After performing regression analysis for determining the impact of age on anemia, the ORs (95% CI) for severe, moderate, and mild anemia were 1.007 (1.004–1.01, *P* < 0.001), 1.011 (1.009–1.013, *P* < 0.001), and 1.013 (1.011–1.016, *P* < 0.001), respectively. The OR for microcytic anemia was 0.984 (0.982–0.985, *P* < 0.001) and for macrocytic anemia it was 1.014 (1.002–1.025, *P*=0.022).

## 4. Discussion

This study was done using the patient's data collected retrospectively for one month from a specialist hospital in Makkah city. In this study, we attempted to analyze the anemia prevalence among the outpatient attendees in various speciality departments. Anemia is a complex disease with nutritional and nonnutritional variables and mechanisms at play. Saudi Arabia, along with the Gulf Arab countries and others, is located in the eastern Mediterranean. In the eastern Mediterranean region, anemia prevalence ranges from 22.6 percent to 63 percent among pregnant women and is 69.6 percent among women of reproductive age [15]. Anemia was found to be prevalent in a high proportion of the patients in this study, according to the findings. The significant prevalence of microcytic anemia in females in the reproductive age range was identified, indicating that iron deficiency is the most likely cause of the condition. It is possible that malnutrition, increased blood loss due to pregnancy or menstruation, and a lack of iron absorption are the primary causes of this condition [6]. Because the serum ferritin levels were not available, we did not corroborate the findings. There have been numerous studies in Saudi Arabia which have revealed a significant frequency of iron deficiency anemia (IDA) in women of reproductive age in the country. IDA is prevalent in 41.6 percent of women between the ages of 18 and 40, according to Alswalem AM [16], and 38.3 percent among Saudi university female medical students, according to another report by Alsheikh [3]. According to the World Health Organization, 2 billion people are anemic worldwide, with iron deficiency accounting for half of all anemia cases. Iron deficiency is the most common micronutrient deficiency in the world [4, 17]

Owaidah et al. [18] described regional variation in the prevalence of iron deficiency and IDA and reported highest prevalence of iron deficiency in the Makkah region. We could not assess the iron deficiency status in our patients wherein IDA appears to be highly prevalent. The overall anemia prevalence reported by AlAssaf [19] was 21% in females and 2.3% in males, whereas AlQuaiz et al. [17] reported anemia prevalence of 40% in women of reproductive age group in Riyadh. The only study reporting low prevalence of 12.5% was done in Tabuk in female university students in the age group of 19–25 years [1]. Majority of the studies conducted in Saudi Arabia have included selected population groups like children below the age of 5, school-going children, young adults/adolescents, pregnant women, and women in reproductive age group. [1, 4, 17, 20–23]. There are regional variations of anemia prevalence in Saudi Arabia within a specific group. Very few studies are conducted in the general population particularly in adults and the elderly [24, 25].

Numerous risk factors for the development of IDA in Saudi women of reproductive age have been identified [4]. Dietary habits, menorrhagia, a history of NSAID use, and a personal or family history of IDA are all risk factors [16, 17, 23, 25]. There is compelling evidence for a negative link between limited meat consumption and an increased risk of developing IDA [3]. As observed in earlier studies, irregular meals and skipping meals, particularly skipping breakfast, are risk factors for IDA [4]. Regular breakfast consumption has an effect on the prevalence of anemia [23]. Vitamin C has been shown to be an effective booster of iron absorption in nonheme meals, and citrus fruits are high in vitamin C [1]. Numerous studies indicate that a family history of genetic disorders is highly associated with IDA, and additional research is needed to confirm this [23]. Another factor for the high incidence is that the Saudi population consumes fewer multivitamin and mineral supplements than many other countries [26]. Microcytic anemia is more prevalent in females due to malnutrition, increased blood loss associated with pregnancy and menstruation, and a deficiency of iron absorption, whereas normocytic anemia is more common in males due to blood loss and chronic disorders [6]. Our results are in agreement with other studies. In our study, the prevalence of anemia in the elderly was 11.68 percent, which is consistent with a report by Alsaeed, who found that the prevalence of anemia in the elderly (>60 years) was 12.9 percent [25]. Males had increased prevalence of anemia with age in our study, whereas females had elevated prevalence in reproductive age groups and a continuous decline in anemia prevalence from the 5th to the 9th decades. Interestingly, normocytic anemia was more widespread in the fifth to ninth decades, demonstrating that anemia is caused by more than iron shortage. Macrocytic anemia was the least reported kind of anemia, accounting for fewer than 2% of cases, which is consistent with data from another Saudi Arabian investigation [13].

The cause of anemia in the senior population must be determined, and fresh studies must be conducted to investigate it. Consumption of tea, which contains a high concentration of polyphenols, has been linked to anemia in the elderly. Polyphenols block nonheme iron absorption. Tea drinking is a widespread ritual in Saudi Arabia and is typically drunk before and after meals [1, 22]. According to a study conducted in China, the high prevalence of anemia among the middle-aged population and elderly was caused by factors such as insufficient consumption of citrus fruits, reduced consumption of red meat, eggs, vegetables, and dairy, and excessive consumption of cereals, cooking oil, and salt, all of which were prevalent among the middle-aged and elderly population in China [16]. Another element to examine is the drastic shift in the Saudi population's food habits from the traditional diet of dates, milk, rice, fresh vegetables, and seafood to junk foods and fewer green vegetables and fruits [20]. Other well-established risk factors for anemia include obesity and malnutrition [20, 27]. Obesity and overweight are considered chronic inflammatory processes and hence play a role in the development of anemia [27]. Anemia is a prevalent disorder among the elderly, and it is connected with an increased risk of death, disability, and impaired physical performance [25]. Numerous factors such as ethnic origin, smoking status, dietary inadequacy, and altitude of residence all influence a person's red cell characteristics, and early detection of anemia in the elderly is critical for rapid intervention and management. The symptoms of anemia are not always obvious in the elderly, and efforts should be taken to determine the most likely cause of anemia. Folate, vitamin B12, serum ferritin, and serum erythropoietin levels should be determined. Additionally, Saudi Arabia has high prevalence of risk factors such as obesity and an unhealthy lifestyle [20].

The World Health Assembly (WHA) has designated anemia reduction as a global dietary priority for 2025 [15]. There is a dearth of research documenting regional progress toward reducing anemia burden and the techniques and interventions being adopted. The trend in anemia prevalence over a ten-year period does not indicate a major decline, and anemia prevalence has remained stable in Saudi Arabia over the last decade [15]. Low and below-average public awareness initiatives can be blamed for the nation's failing health [22]. Saudi Arabia should take a multisectoral, community-based strategy to prevent and control anemia. Along with dietary inadequacies, nonnutritional causes of anemia such as acute and chronic parasite infections and genetic illnesses such as thalassemia, G6PD deficiency, and sickle cell trait must be addressed [15].

### 4.1. Limitations

Our study has some limitations. We included patients who visited a hospital, which may have inflated the numbers, but we excluded hospitalized patients. However, our findings are consistent with those of other research conducted in Saudi Arabia.

## 5. Conclusion

Our study showed high prevalence of anemia among the patients attending outpatient departments in a specialist hospital. Females have high prevalence of anemia when compared to male population. Microcytic anemia was the most common anemia type among females and was prevalent in the 15–49 age group considered as the reproductive age group, whereas normocytic anemia was more common among the male gender. Anemia of mild and moderate severity was predominant in both genders, although severe anemia showed higher prevalence in females as compared to males. The authorities need to address the problem of prevention and reduction in anemia prevalence in Saudi Arabia by taking effective measures and interventions.

## Figures and Tables

**Table 1:** Hemoglobin levels for stratification into mild, moderate, and severe anemia [14].

	Nonanemia	Anemia		
		Mild	Moderate	Severe
Nonpregnant women (15 years of age and above)	120 or higher	110–119	80–109	<80
Men (15 years of age and above)	130 or higher	110–129	80–109	<80

**Table 2:** Hematological variables and demographic data.

Variable	Mean	SD	Minimum value	Maximum value	Median
*Age (years)*					
Male	48.84	18.17	15	89	46
Female	46.88	17.8	15	88	47

*Hb level (g/dl)*					
Male	100.12	19.2	3.12	21.5	14.3
Female	98.8	18.2	2.56	23.3	12.1

*Mean cell volume (µm* ^*3*^)					
Male	83.86	6.56	47.8	138.9	84.5
Female	81.77	7.80	46.6	120.6	82.88

*MCH (pg)*					
Male	28.08	2.71	12.3	54.6	28.58
Female	26.65	3.27	13.15	59.60	27.3

*MCHC (g/dl)*					
Male	32.4	38.12	8.26	64.3	33.35
Female	31.3	39.57	7.57	70.5	32.45

*RDW (%)*					
Male	14.13	2.03	10.9	35.5	13.6
Female	14.91	2.31	11	32.7	14.28

*RBC (m/µl)*					
Male	5.05	0.75	1.35	10	5.12
Female	4.54	0.56	1.75	7.43	4.56

**Table 3:** Severity of anemia across age groups in males.

			Age groups								Total
			≤19	20–29	30–39	40–49	50–59	60–69	70–79	>80	
HGB categories	Severe anemia	Count	29	64	65	45	55	73	48	25	404
		% of total	1.1%	2.4%	2.5%	1.7%	2.1%	2.8%	1.8%	0.9%	15.2%
	Moderate anemia	Count	59	126	130	119	159	205	141	102	1041
		% of total	2.2%	4.8%	4.9%	4.5%	6.0%	7.7%	5.3%	3.8%	39.3%
	Mild anemia	Count	68	100	129	118	202	273	210	105	1205
		% of total	2.6%	3.8%	4.9%	4.5%	7.6%	10.3%	7.9%	4.0%	45.5%

Total		Count	156	290	324	282	416	551	399	232	2650
		% of total	5.9%	10.9%	12.2%	10.6%	15.7%	20.8%	15.1%	8.8%	100.0%

**Table 4:** Severity of anemia across age groups in females.

			Age groups								Total
			≤19	20–29	30–39	40–49	50–59	60–69	70–79	>80	
HGB categories	Severe anemia	Count	48	146	176	201	149	116	92	49	977
		% of total	0.8%	2.6%	3.1%	3.5%	2.6%	2.0%	1.6%	0.9%	17.2%
	Moderate anemia	Count	117	346	527	536	370	375	225	120	2616
		% of total	2.1%	6.1%	9.3%	9.4%	6.5%	6.6%	4.0%	2.1%	46.0%
	Mild anemia	Count	91	353	371	388	356	303	157	77	2096
		% of total	1.6%	6.2%	6.5%	6.8%	6.3%	5.3%	2.8%	1.4%	36.8%

Total		Count	256	845	1074	1125	875	794	474	246	5689
		% of total	4.5%	14.9%	18.9%	19.8%	15.4%	14.0%	8.3%	4.3%	100.0%

**Table 5:** Prevalence of morphological types of anemias across age groups in males.

			Age groups								Total
			≤19	20–29	30–39	40–49	50–59	60–69	70–79	>80	
MCV groups	Microcytic	Count	109	167	159	142	182	260	146	84	1249
		% of total	4.1%	6.3%	6.0%	5.4%	6.9%	9.8%	5.5%	3.2%	47.1%
	Normocytic	Count	47	123	158	135	234	286	249	145	1377
		% of total	1.8%	4.6%	6.0%	5.1%	8.8%	10.8%	9.4%	5.5%	52.0%
	Macrocytic	Count	0	0	7	5	0	5	4	3	24
		% of total	0.0%	0.0%	0.3%	0.2%	0.0%	0.2%	0.2%	0.1%	0.9%

Total		Count	156	290	324	282	416	551	399	232	2650
		% of total	5.9%	10.9%	12.2%	10.6%	15.7%	20.8%	15.1%	8.8%	100.0%

**Table 6:** Prevalence of morphological types of anemias across age groups in females.

			Age groups								Total
			≤19	20–29	30–39	40–49	50–59	60–69	70–79	>80	
MCV groups	Microcytic	Count	202	629	824	857	540	419	215	98	3784
		% of total	3.6%	11.1%	14.5%	15.1%	9.5%	7.4%	3.8%	1.7%	66.5%
	Normocytic	Count	53	209	245	261	325	369	254	144	1860
		% of total	0.9%	3.7%	4.3%	4.6%	5.7%	6.5%	4.5%	2.5%	32.7%
	Macrocytic	Count	1	7	5	7	10	6	5	4	45
		% of total	0.0%	0.1%	0.1%	0.1%	0.2%	0.1%	0.1%	0.1%	0.8%

Total		Count	256	845	1074	1125	875	794	474	246	5689
		% of total	4.5%	14.9%	18.9%	19.8%	15.4%	14.0%	8.3%	4.3%	100.0%

## Data Availability

Our conclusions arise from the evaluation of the demographic and laboratory data accessed from the hospital records and are described in this study. These data cannot be released due to patient confidentiality.
